# A qualitative exploration of the factors associated with initiation to methamphetamine use in Iran

**DOI:** 10.1186/s12889-020-09908-7

**Published:** 2020-11-23

**Authors:** Shirin Shahbazi Sighaldeh, Fatemeh Zarghami, Ali Shahryari, Ali Mohammadinia, Mohsen Ebrahimi, Teimoor Jorjani, Mohammad Shoaib Hamrah, Abdurrahman Charkazi

**Affiliations:** 1 Reproductive Health Department, School of Nursing and Midwifery, Tehran University of Medical Sciences, Tehran, Iran; 2 Department of Biostatistics and Epidemiology, Department of Health Education and Health Promotion, Faculty of Health, Tabriz University of Medical Sciences, Tabriz, Iran; 3 Environmental Health Research Center, Faculty of Health, Golestan University of Medical Sciences, Gorgan, Iran; 4 Public Health, Golestan University of Medical Sciences, Gorgan, Iran; 5 Neonatal and Children’s Health Research Center, Golestan University of Medical Sciences, Gorgan, Iran; 6 Shahid Motahari Hospital, Golestan University of Medical Sciences, Gonbad-e-Qabous, Iran; 7 Center for Rural Health, School of Health Sciences, University of Tasmania, Hobart, Australia; 8 Health Education and Promotion, Environmental Health Research Center, Faculty of Health, Golestan University of Medical Sciences, Late Falsefi University Complex, KM 5of Gorgan-Sari Road, Golestan, Iran

**Keywords:** Crystal, Methamphetamine, First use, Qualitative study

## Abstract

**Background:**

Crystal (methamphetamine) is a strong stimulant of addictive substances that affects the central nervous system. The consumption of this substance is increasing among teenagers and adult young people in the country. In this matter, one of the practical and important ways to its control is to identify the factors leading to its first use. Thus this paper, explores the factors related to the first crystal use in Golestan province, Iran.

**Methods:**

In a qualitative study, 19 crystal users were recruited in the study conducted in Golestan province by snowball sampling from DIC (Drop-In Center) in addiction treatment centers. The interviews were mostly carried out individually with the participants; only one interview was conducted in an addiction treatment camp in the form of a group-focused discussion. Data analysis was implemented through content analysis in MAXQDA 10 software.

**Results:**

The mean age of the participants was 35.05 ± 6.06 years with a range of 23–46 years. Meanwhile, the mean of crystal use period was 7.42 (SD: 3.61) and a range of 1–14 years. Based on the obtained data, the reasons for first crystal use could be categorized in six; 1: “crystal use to other drugs rehabilitation”; 2: “Lack of awareness of the addictive nature of crystal”; 3: “stimulating curiosity in public with crystal-use friends”, 4: affordable and convenient use”, 5: “anti-sleeping effects and increasing work efficiency”, and 6: “therapeutic and other misconceptions”“.

**Conclusions:**

The results indicated that crystal is mostly abused to opium rehabilitation. The lack of awareness and misconceptions about this substance can lead people to crystal use. Therefore, designing public health interventions to increase awareness about negative consequences of crystal use is fundamental to prevent people from abusing it. We suggest designing public health program to promote awareness about health risks of crystal and modifying related misconceptions. Finally, the government can establish policies to mandate sale tax for crystal producers and reduce easy access to crystal especially among youth.

## Background

In the early twentieth century, the amphetamines and their derivatives were first abused as bronchodilators [1]. In this regard, the methamphetamine (crystal) is a strong stimulant of addictive substances that affects the central nervous system, which is easily produced in underground laboratories [2, 3]. The acute effects of methamphetamine are related to increased energy and attention, increased positive mood, and decreased appetite [4].

The half-life of methamphetamine is approximately long (8–12 h), compared to other stimulant substances such as cocaine and nicotine. Here, it should be mentioned that this substance is a central nervous system stimulant; it facilitates the release of Norepinephrine and Dopamine from Neurotransmitter and delays their re-absorption to some extent [5]. Besides, the methamphetamine deprives tissues of oxygen by vasoconstriction, while the high norepinephrine circulation can lead to tissue damage [6, 7]. Moreover, the continued use of methamphetamine can damage various body organs. In this regard, the heart conditions such as chest pain, arrhythmia, hypertension, cardiomyopathy and even heart attack have noticeably been observed in young consumers [7–9]. The results of a conducted study of 353 methamphetamine consumers who went to the emergency department indicated that about 18.7% of them, had mental health problems, 18.4% suffered from trauma, 11.11% suffered skin infections, and 9.6% suffered from oral problems [10]. At the meantime, the heart attack and seizures have also been reported in methamphetamine consumers [11–13].

Other problems such as movement problems such as hyperkinetic movements, the stereotyped and repetitive behaviors may also be observed [14, 15]. In this matter, recent studies confirmed the relationship between methamphetamine use and severe dental disease, known as “meth mouth” [16, 17]. Other studies reported that about 40% of methamphetamine consumers have experienced complete neurodegenerative psychological damage [18]. Here, the crystal use would cause tolerance and dependency that leads to creating a condition called psychosis methamphetamine or paranoid schizophrenic [19].

The relevant researches have shown that crystal use is rising among teens and young people around the world, which can lead to complications such as pathology, mental retardation, auditory hallucinations, suicidal ideation, depression, anxiety, violence, fatigue, suspicion, aggression, severe temptation to usage, high-risk sexual behaviors, cardiovascular problems and even death [20, 21].

In this way, a 2015 UN report revealed that the production of stimulant substances has been increased from 34 tons in 2009 to 88 tons in 2013 [21], which this is probably why the Iranian studies have reported that in recent years, the usage pattern has been changed from the traditional substances to industrial and the usage type has been converted from tobacco to injection. In 2008, more than 6% of drug addicts over the age of 12 in Iran were crystal consumers, while most of them were young people. Hence, the amount of crystal use has dramatically increased from 2008 to 2012. A conducted study in 2010 by praying people found that 25% of drug addicts were crystal consumers, in which the average age of them was 18 years [22, 23]. The results of an implemented study in the country showed that 15% of Iranian addicts abuse the crystal [24]. The results of a study in Zahedan also confirmed that the amount of crystal use among people referring to a methadone maintenance center has been increased from 6% in 2009 to 20% in 2011 [25]. To succeed in the addiction prevention programs, it is essential to identify the main causes of people’s addiction and its contributing factors. In the meantime, the qualitative studies can provide vital and valuable information to the researcher. For this reason, to determine the main causes of people’s tendency to crystal abuse, such (qualitative) studies can provide access to the real conditions of people and their experience. They can gather data without any change or intervention and prepare an accurate reflex of the main causes of the crystal use [26].

Here, it should be noted that methods based on questionnaires and closed-ended questions that are commonly employed, may restrict the depth of participants’ answers, and as a result, the quality of the data may be either reduced or incomplete. As such, the questionnaire-based methods, where there is little or no information on an issue or area of ​​content, are not the proper method to choose. In such cases, the qualitative methods are strongly better [27]. Regarding the fact that the number of qualitative, in-depth and comprehensive studies on the causes of crystal use in our country is limited to obtain comprehensive, in-depth and first-hand information, this paper was conducted in Golestan province, Iran to explore the main factors related to the first usage of crystal.

## Methods

This qualitative study conducted 13 in-depth interviews and 6 focus groups to elicit information on people’s motivations and experiences in using methamphetamines for the first time. Interviews were conducted between June and December 2019, and total of 19 participants were recruited.

In this qualitative study, which was conducted in 2019 on 19 participants from different cities of the province who either have been consuming the crystal for at least 6 months or have previously consumed it for the same period? These participants were chosen from Drop In Center (DIC), addiction treatment camps, and the location of the crystal addicts. After describing the aims of the study and obtaining informed consent, they entered the study. In one of the addiction camps, the interview was conducted in the form of a group discussion (6 people). The rest of the interviews were conducted individually and in a comfortable and safe place at the choice of the participant (camp interview room and DIC centers). To collect data, the in-depth semi-structured interviews were utilized. The interview time averaged 45 min. The interviews with the permission of the participants recorded and then transcribed word-by-word. Interview questions were asked with the help of an interview guide (supplementary file 1). In addition, before starting any interview, all participants completed the social demographic specifications questionnaire and the crystal consumption pattern. Then, the participants were asked to share their experiences about people’s tendency to initiate crystal use, and then were asked to focus on expressing his/her personal experiences about the main reasons that led to his/her desire to abuse the crystal. The next questions will be designed based on both people’s initial answers and interview guidance. Moreover, based on the need, in interviews, some exploratory questions such as “What do you mean?” Or “If you can please explain more”, employed. At the end of each interview, the participant was asked to speak slowly if he/she spoke, and then to talk to him or her about the possibility of further interviews. The sampling continued until saturation. In this paper, 14 participants, the study went to saturation stage, but to ensure this, the interviews continued until the 19th person. The interviews finished due to the repetition of conversations and the lack of new content added to the previous interview content. Written informed consent was obtained from all participants.

To analyze the gathered data, we transcribed the audio interface in the form of a secret scripture, followed by a careful study of the scriptures, which are the same units of analysis that were attempted to get a general sense of them. Then the Lundman’s and Grenheim’s methods were performed for analysis for organizing the text of the interviews with open source code [28]. The extracted code management was carried out based on MAXQDA10 text data organization software.

## Results

The average age of the participants was 35.05 (SD: 6.06, IQ Range: 9) years with age range of 23–46 years. The mean of crystal consumption period was 7.42(SD: 3.61) with a range of 1–14 years. In addition to the crystal, all participants mentioned the simultaneous use of other substances with 15 (78.9%) participants smoking cigarette as the most common simultaneous substance. Analyzing the demographic profile of the participants showed that 9 participants (47.3%) had primary school education, 5 (26.3%) had secondary school education, and 3 (15.7%) were high school dropouts. Were ethnically diverse, and were unemployed or working (as listed in Table 1). According to the qualitative data, the reasons for the first crystal use were categorized in six factors. These factors are listed in Table 2.

**Table 1:** Study participant profile

Participant ID	Crystal addiction period (years)	Other drug use history	Education	Ethnicity	Place of residence
1	13	Cigarette,Opium,Hashish,Tamjizac	University degree	Persia	Gorgan
2	14	Methadone,Opium, Heroin,Crack, Cigarette	Primary school	Persia	Aq Qala
3	8	Methadone,Opium, Crack, Cigarette	High school dropout	Turkmen	Aq Qala
4	10	Opium, Heroin, Crack,	High school dropout	Turkmen	Aq Qala
5	3	Opium, Cigarette	Primary school	Persia	Bandar Gaz
6	4	Opium, Cigarette	Secondary school	Persia	Bandar Gaz
7	10	Opium, Cigarette, Crack	Primary school	Turkmen	Aq Qala
8	10	Opium, Cigarette, Crack	Primary school	Persia	Ali Abad
9	12	Opium, Cigarette, Crack	Secondary school	Turkmen	Aq Qala
10	7	Opium, Cigarette, methadone	Secondary school	Turkmen	Ghonbad
11	4	Opium, Crack	Primary school	Turkmen	Ghonbad
12	6	Opium, Cigarette	Primary school	Turkmen	Ali Abad
13	8	Opium, Cigarette	High school diploma	Persia	Azadshaher
14	8	Alcohol,Methadone,Opium, Heroin	Secondary school	Turkmen	Ghonbad
15	8	Cigarette, Methadone	Primary school	Persia	Ghonbad
16	8	Opium	Secondary school	Balouch	Azadshaher
17	4	Opium, Cigarette	Primary school	Balouch	Azadshaher
18	1	Opium, Cigarette, Heroin	Primary school	Persia	Azadshaher
19	3	Opium, Cigarette, Heroin	High school dropout	Persia	Azadshaher

**Table 2:** Categories of the factors associated with initiation into the crystal use

Category #	Description of Category
**1**	Crystal use to other drugs rehabilitation
**2**	Lack of awareness of the addictive nature of crystal
**3**	Stimulating curiosity in public with crystal-user friends
**4**	Affordable and convenient use
**5**	Anti-sleeping effects and increasing work efficiency
**6**	Therapeutic and other misconception

### Crystal usage to other drugs rehabilitation

Most participants (14 people) mentioned morphine as the reason for the crystal use. In other words, they presumed that by consuming the crystal, they could rehabilitate the addictive drugs containing morphine, such as opium, crack, and heroin. They falsely believed that crystal does not have hangover and body pain.

*“I went for it because of a dirty thought; I meant a dirty thought that I was thinking of leaving crack and I heard that crystal doesn't have morphine and that's why I went to abuse it." (Participant #3)*

### Lack of awareness of the addictive nature of crystal

A large number of participants (12 people) believed that the crustal was not addictive and its consuming doesn’t contain the hangover of morphine, and that is why they started abusing it. In Iran, drug dealers are called “butlers”. The butlers told the studied participants that the crystal did not cause any addiction or dependence. On the other hand, the participants also believed that the crystal did not cause physical dependence and that if you wanted to leave it, it would not be physically painful. However, after abusing it, they realized that although the crystal does not cause any physical dependence, it severely affects the consumer psychologically.

*“The butler told us that a new drug has arrived that is very fun and not addictive. I said it's addictive!! Because we considered addiction to be a pain in the body, and we thought that a substance that causes pain in the body is addictive, then our way of thinking was like this, and we were not at all familiar with mental and psychological hangover. We considered hangover to be physical, while the crystal is not physically hangover, and the body has no pain when it breaks. We were told to abuse these substances, which gives them a good mood and makes them feel weird, and we did that.” (Participant #4)*

### Stimulating curiosity in public with crystal-usage friend

Curiosity and observing the crystal usage by friends, introducing it, and their compliments have caused a number of participants (4 people) to mention it as the main reason for the first crystal use. Curiosity intimate young people to try new drugs when they enter the consumer market.

*“We were sitting in a group. The crystal had just arrived and we were curious what it was. When they complimented us, we abused it.” (Participant #6).*

*“Then, I saw how many people were consuming and enjoying it. Then, one of my friends said, "Give it a try, but I said no and I didn't." But after a while we went there, I wanted to know what it was, and I abused it.” (Participant #15).*

### Affordable and convenient use

The participants believed that the price of crystal was so cheap that it could be easily paid for. In addition, the comfortable and easy usage of crystal is one of its advantages over other drugs, so that they can abuse it with just a small pipe and a match or lighter wherever they want.

*“The addict population, e.g. myself, see the cheaper substance, the more it definitely abuses. With this market situation, most young people are moving towards abusing it, because it is both easy to abuse and comfortable, while its price is suitable.” (Participant #1)*

*“Yes, they said that these substances are good substances and there is no hangover. It is very cheap and very easy to abuse. Pour it into a small pipe, and the lighter inside the pocket, wherever we want to go, even in the bathroom, we can easily abuse it. We light a match under it and then consume it.” (participant number#5)*

### Anti-sleeping effects and improved work efficiency

Another reason for the first consumption of crystal which was mentioned by 7 participants (36.8%) was its anti-sleeping effects, and the increased efficiency of work. They only wanted to test the crystal for the first time and take advantage of its potential to help them stay awake, but this usage continued after the first time. People who need to stay awake at night or those who are looking for more energy in work are among those who abuse the crystal.

*“I ate methadone and then fell asleep while guarding. One day, I was at the house of one of my friends, I was making nonsense, he was also consuming industrial substances and he said that if you abuse this, you would not sleep. Then I told myself to abuse it once to see how it is!! I tried it once and then it continued on.” (Participant #13)*

*“Yes. I was told to abuse it, because my work is hard. My friend told me to come here and abuse it once. If you abuse the crystal, your work efficiency will be increased. If you work 12 hours now, you can work 18 hours. If you work 18 hours, you can work 20 hours. Early on, I was able to work the same amount of time, but later it didn't take more than an hour.” (Participant #9)*

### Therapeutic and other misconception

A number of participants had misconceptions about crystal that caused them to the first usage. One of them stated that the reason to abuse the crystal was to eliminate the kidney stone and believed that the crystal use helps to excrete the kidney stones:

*“I had kidney stones and worked somewhere as a laborer. I asked one of the kids who worked there how it was that you worked for three days and went to work again. He said that I would abuse the crystal and this would reduce the body water and I could not work anymore. Then I said that this should be good for kidney stones.” (Participant #12)*

A few people also believed that as they heard that the crystal usage can increase the sexual pleasure and affects the beauty of appearance and face, they started abusing it.

*“My friend was abusing it and I didn't know. His first words it was good for sex and beauty. At first, it was talk, and student spending was high. Most doctors abused it to wake him up. Now I have nothing to do with advertising. For these three reasons, I also started abusing it.” (Participant #16).*

## Discussion

This study was conducted to explore the main factors leading to the first consumption of crystal in Golestan province, Iran. The Results of this study revealed that these factors could be divided into six different categories.

### Crystal use to other drugs rehabilitation

One of the most important reasons for the first crystal use was to rehabilitate other drugs. All participants in the study were simultaneously addicted to other drugs before addiction to crystal and they have misconceptions that that they could not abuse other substances such as crack, heroin and opium by abusing the crystal.

Sometimes, this misconception has been proposed by drug dealers trying to convince people so they can sell their drugs. Another factor influencing this misconception has been the promotion and recommendation of friends addicted to crystal. This misconception stems from the fact that crystal consumers are more likely to emphasize the physical dependence on opium, and their perception is more of the physical symptoms of quitting opium such as crack and heroin.

While the crystal use makes it tolerable, which causes a condition so-called psychosis methamphetamine, which is a paranoid schizophrenia-like condition [19]. The results of a study conducted by Noori et al. confirmed that the tendency to opium rehabilitation is the most important reason for tendency to abuse the crystal [29]. Furthermore, due to the tolerance phenomenon caused by abusing some previous substances such as heroin and crack, it seems that people start abusing novel substances such as crystal. The results of a study indicated that reducing the effects of the previous abused substances was the most important reason to switch from previous substance into crystal [30].

### Lack of awareness of the addictive nature of crystal

The belief in the non-addiction nature of crystal which was mentioned by a number of consumers was one of the reasons leading to the first use of crystal. Lack of information about the addictive characteristics of crystal can be considered as one of the important causes of this addiction. Bostanizadeh and Karami investigated women prisoners addicted to crystal in Iran. They described issues such as non-addictiveness of crystal, heroin and crack, weight loss and obesity as interfering factors that facilitate the crystal use [31].

### Stimulating curiosity in public with crystal-use friends

Curiosity with peer pressure has always been one of the most important reasons to start abusing a variety of substances with crystal being no exception. The results of the conducted study by Sahami and Kazemi using the Grounded Theory revealed that to abuse the crystal, joining crystal-abusing friendship groups creates a tendency to abuse. At first, the usage motivation is raised by curiosity or recreation, but over time, sterile beliefs about addiction are established in the individual, which leads to coercion, voracity, and diversity of abusing [32]. Another study confirmed that curiosity is one of the causal factors in crystal abuse [31].

### Affordable and convenient use

A number of participants mentioned the low price and ease of abusing it as the reason for the first usage of crustal. Regarding the abundant production of crystal in Golestan province, which is well known as a central kitchen among consumers, the term of its abuse has increased, and therefore, it has overcome the demand and reduced its price, as compared to previous years. On the other hand, it makes it easier to access. These two factors, represented as the facilitators, can prepare the stage for the crystal usage. On the other hand, its easy consumption is one of the reasons for the first usage. Abusing some traditional substances such as opium and juice requires special time, organization and equipment. However, the crystal can easily be consumed via a pipe and lighter in a few minutes. Meanwhile, there is no special smoke and smell produced. However, the time of abusing the opium is very long compared to crystal, and due to the smell and smoke caused by smoking; it can be accompanied by a lot of attention. In a study conducted by Yoosefi Lebni et al. it is reported that easy access and easy use were the main reasons of Crystal abuse [33].

### Anti-sleeping effects and improved work efficiency

The anti-sleep effects and increasing work efficiency were mentioned by some of the participants in this study as reasons for the first use. Note that as the crystal is a class of amphetamines and is a stimulant, it can lead to insomnia and prolonged awakening, as well as increased energy. It would lead creating an overnight that people turn around it to increase the work efficiency. The participants in the study, who mentioned this factor, said they had either seen it with their own eyes among their friends, or that the effects had been passed on to them, thus encouraging the crystal usage among them. In one study, the tendency to increase performance and efficiency was mentioned as one of the important reasons for the first of crystal use [29]. Therefore, these days crystal is being abused by drivers and other groups with overnight shifts to increase their awareness and performance [30].

### Therapeutic and other misconception

Misconceptions such as the kidney stones disease treatment, increasing sexual pleasure, and facial beauty were other reasons in initiation of crystal use. Aesthetic issues such as facial beauty and weight loss in both studies have been described as reasons for the tendency for the first usage of crystal [29, 31]. Nevertheless, the subject of medical treatments has previously been more concerned with opium usage [34]. However, in the current research, one of the participants mentioned belief in the treatment of kidney stones.

## Conclusion

The results of this paper indicated that morphine rehabilitation, the lack of awareness (non-addictiveness of crystal), curiosity, compliments of friends, cheap price and comfortable use, anti-sleeping effects and increasing work efficiency, and misconceptions were the most important reasons leading to the first crystal use. We suggest designing public health program to promote awareness about health risks of crystal and modifying misconceptions about crystal use. Finally, the government authorities should make policies to obtain mandatory sale tax from crystal producers and reduce easy access to crystal especially among younger people.

## Supplementary Information

**Additional file 1.** Interview guide; questions which used and asked from participants.

## Data Availability

The data are available from corresponding author; Abdurrahman Charkazi from reasonable request.

## Abbreviations

IRB: Institutional Review Board
DIC: Drop-In-Center

## References

[1] Richards JR, Hamidi S, Grant CD, Wang CG, Tabish N, Turnipseed SD, et al. Methamphetamine use and emergency department utilization: 20 years later. J Addict. 2017;2017. 10.1155/2017/4050932. 10.1155/2017/4050932 PMC5585625 28913001

[2] Baskin-Sommers A, Sommers I (2006). Methamphetamine use and violence among young adults. J Crim Just.

[3] Herman-Stahl MA, Krebs CP, Kroutil LA, Heller DC (2007). Risk and protective factors for methamphetamine use and nonmedical use of prescription stimulants among young adults aged 18 to 25. Addict Behav.

[4] Rawson RA, Gonzales R, Brethen P (2002). Treatment of methamphetamine use disorders: an update. J Subst Abus Treat.

[5] King GR, Ellinwood E (1997). Amphetamines and other stimulants. Substance abuse A comprehensive textbook 3th edition Baltimore: Williams & Wilkins.

[6] Karch SB, Drummer O (2008). Karch's pathology of drug abuse: CRC press.

[7] Kaye S, McKetin R, Duflou J, Darke S (2007). Methamphetamine and cardiovascular pathology: a review of the evidence. Addiction..

[8] Turnipseed SD, Richards JR, Kirk JD, Diercks DB, Amsterdam EA (2003). Frequency of acute coronary syndrome in patients presenting to the emergency department with chest pain after methamphetamine use. J Emerg Med.

[9] Ujike H, Sato M (2004). Clinical features of sensitization to methamphetamine observed in patients with methamphetamine dependence and psychosis. Ann N Y Acad Sci.

[10] Hendrickson RG, Cloutier R, John MCK (2008). Methamphetamine-related emergency department utilization and cost. Acad Emerg Med.

[11] Perez JA, Arsura EL, Strategos S (1999). Methamphetamine-related stroke: four cases. J Emerg Med.

[12] Richards JR, Bretz SW, Johnson EB, Turnipseed SD, Brofeldt BT, Derlet RW (1999). Methamphetamine abuse and emergency department utilization. Western J Med.

[13] Westover AN, McBride S, Haley RW (2007). Stroke in young adults who abuse amphetamines or cocaine: a population-based study of hospitalized patients. Arch Gen Psychiatry.

[14] Mattson RH, Calverley JR (1968). Dextroamphetamine-sulfate-induced dyskinesias. JAMA..

[15] Sperling LS, Horowitz JL (1994). Methamphetamine-induced choreoathetosis and rhabdomyolysis. Ann Intern Med.

[16] Curtis E (2006). Meth mouth: a review of methamphetamine abuse and its oral manifestations. Gen Dent.

[17] Glasner-Edwards S, Mooney LJ, Marinelli-Casey P, Hillhouse M, Ang A, Rawson RA (2010). Psychopathology in methamphetamine-dependent adults 3 years after treatment. Drug Alcohol Rev.

[18] Rippeth JD, Heaton RK, Carey CL, Marcotte TD, Moore DJ, Gonzalez R (2004). Methamphetamine dependence increases risk of neuropsychological impairment in HIV infected persons. J Int Neuropsychol Soc.

[19] Iritani BJ, Hallfors DD, Bauer DJ (2007). Crystal methamphetamine use among young adults in the USA. Addiction..

[20] Rawson RA, Gonzales R, Obert JL, McCann MJ, Brethen P (2005). Methamphetamine use among treatment-seeking adolescents in Southern California: participant characteristics and treatment response. J Subst Abus Treat.

[21] Drugs UNOo, Crime (2010). World drug report 2010: United Nations Publications.

[22] Doaghoyan D, Habibzadeh MA (2011). J Disciplinary Knowledge.

[23] Bashirian S, Hidarnia A, Allahverdipour H, Hajizadeh E (2012). Application of the theory of planned behavior to predict drug abuse related behaviors among adolescents. 2012. JRHS.

[24] Shekarchizadeh H, Ekhtiari H, Khami MR, Virtanen JI (2012). Patterns of pre-treatment drug abuse, drug treatment history and characteristics of addicts in methadone maintenance treatment in Iran. Harm Reduct J.

[25] Lashkaripour K, Yusefi M, Ghasemi S, Zabihi R. The comparison of demographic characteristics and variety of substances at methadone maintenance clinic of Baharan psychiatry hospital in 2009–2010: Abstract book of Fifth Addiction congress in Zahedan; 2012.

[26] Woods P (2006). Successful writing for qualitative researchers: psychology press.

[27] Rattray J, Jones MC (2007). Essential elements of questionnaire design and development. J Clin Nurs.

[28] Graneheim UH, Lundman B (2004). Qualitative content analysis in nursing research: concepts, procedures and measures to achieve trustworthiness. Nurse Educ Today.

[29] Noori R, Daneshmand R, Farhoudian A, Ghaderi S, Aryanfard S, Moradi A. Amphetamine-type stimulants in a group of adults in Tehran, Iran: a rapid situation assessment in twenty-two districts. Iran J Psychiatry Behav Sci. 2016;10(4). 10.17795/ijpbs-7704.

[30] Barati M, Allahverdipour H, Moeini B, Farhadi NA, Mahjub H, Jalilian F (2011). Assertiveness skills training efficiency on college students’persuasive subjective norms against substance abuse. Avicenna J Clin Med.

[31] Boostani D, Karamizadeh E (2017). Conditions and strategies of crystal (Methamphetamine) consumption among addicted women (Case study: Kerman City). J Women Dev Polit.

[32] Sahami S, Khezri Z (2014). Conceptual Model of Metaphetamine Misusage. J Psychol Models Methods.

[33] Lebni JY, Ziapour A, Qorbani M, Khosravi B, Mirzaei A, Safari O, et al. Explaining the causes of crystal addiction in Tehran: a qualitative approach. J Public Health. 2019:1–7. 10.1007/s10389-019-01093-1.

[34] Jafari S, Movaghar AR, Craib K, Baharlou S, Mathias R (2009). Socio-cultural factors associated with the initiation of opium use in Darab, Iran. Int J Ment Heal Addict.

