# Supplementary material for: A qualitative exploration of the factors associated with initiation to methamphetamine use in Iran
Source: BMC Public Health. 2020 Nov 23;20:1773. doi: 10.1186/s12889-020-09908-7 (PMC7684954; doi:10.1186/s12889-020-09908-7)
Supplement: Supplementary file 1 — Additional file 1. Interview guide; questions which used and asked from participants. [file 12889_2020_9908_MOESM1_ESM.docx]

| **Interview guide** | |
| --- | --- |
| **Questions #** | **Interview questions** |
| **1** | From your point of view, why some people start using crystal? |
| **2** | When was the first time you start using crystal smoking? Why did you start it? |
| **3** | Please explain more about your first experience in using crystal? Where was it and who accompanied you in this experience? |
| **4** | What were other reasons for initiation of crystal use? |
| **5** | You are welcome to add to what you provided on your crystal using experience. |
